# A Case Report of Drug‐Induced Hemorrhagic Bullae

**DOI:** 10.1002/ccr3.70423

**Published:** 2025-04-15

**Authors:** Mehrdad Shavandi, Zohre Labbani‐Motlagh, Azin Abdollahi, Hadieh Tazerouni, Alireza Naserian, Shahideh Amini

**Affiliations:** ^1^ Rajaie Cardiovascular Medical and Research Institute Tehran Iran; ^2^ Toxicology Research Center Aja University of Medical Sciences Tehran Iran; ^3^ Faculty of Pharmacy Tehran University of Medical Science (TUMS) Tehran Iran; ^4^ Faculty of Medicine, Department of Internal Medicine Tehran University of Medical Science (TUMS) Tehran Iran

**Keywords:** adverse drug reaction, Alteplase, hemorrhagic bullae, polypharmacy, vancomycin

## Abstract

Hemorrhagic bullae is a rare, non‐immune, cutaneous adverse reaction that happens after the administration of some medicine. This case is a unique report of vancomycin and alteplase‐induced hemorrhagic bullae and warns physicians and healthcare teams to take action properly.

## 1 Introduction

Hemorrhagic bullae is a rare skin reaction that can occur due to various causes. One potential cause is drug‐induced hemorrhagic bullae, which can result from medications such as warfarin and heparin. However, there are no reports of hemorrhagic bullae due to the administration of thrombolytic or most of the antibiotics. While polypharmacy can increase the risk of adverse reactions, there have been no previous reports of hemorrhagic bullae induced by polypharmacy.

Alteplase (recombinant tissue plasminogen activator, r‐TPA) as a thrombolytic agent is primarily indicated in patients suffering from acute ST‐segment elevation myocardial infarction (STEMI), acute ischemic stroke, and pulmonary embolism. The most common adverse events of thrombolytic therapy include intracerebral hemorrhage and gastrointestinal and genitourinary hemorrhage [1, 2]. Hemorrhagic bullae secondary to thrombolytics are not well recognized yet.

Vancomycin is a glycopeptide antibiotic and is used in the treatment of Gram‐positive bacterial infections. The most common adverse events of vancomycin include ototoxicity, hypersensitivity reactions, nephrotoxicity, and gastrointestinal symptoms. Also, one of the rare adverse reactions of vancomycin is linear IgA bullous dermatosis [3].

Meropenem is a carbapenem antibiotic and is used in the treatment of Gram‐positive and Gram‐negative, and anaerobic bacterial infections. The most common adverse events of vancomycin include nausea and vomiting, diarrhea or constipation, and inflammation at the injection site. However, there is no report of meropenem‐induced hemorrhagic bullae [4].

We report a case of hemorrhagic bullae caused by systemic polypharmacy in an elderly patient who suffered a cerebrovascular accident (CVA).

This is a unique case of hemorrhagic bullae following the administration of multiple medications, alerting physicians and the healthcare team about the possibility of this adverse reaction occurring with polypharmacy.

## 2 Case History/Examination

A 68‐year‐old man was admitted to the emergency department (ED) at Shariati Hospital with a sudden history of right‐sided hemiparesis, aphasia, fever, and gradual loss of consciousness. He was taken to the hospital as soon as his family noticed the symptoms. His medical history was significant for hypertension, diabetes, chronic kidney disease, and a prior coronary artery bypass graft surgery. The current medication includes atorvastatin, carvedilol, aspirin, and allopurinol. There has not been any change in his medication recently.

A general physical examination revealed an oral temperature of 38.2°C, a heart rate of 90 beats per minute, a respiratory rate of 14 breaths per minute, a blood pressure of 150/86 mmHg, and an oxygen saturation of 94% with room air.

**FIGURE 1:**
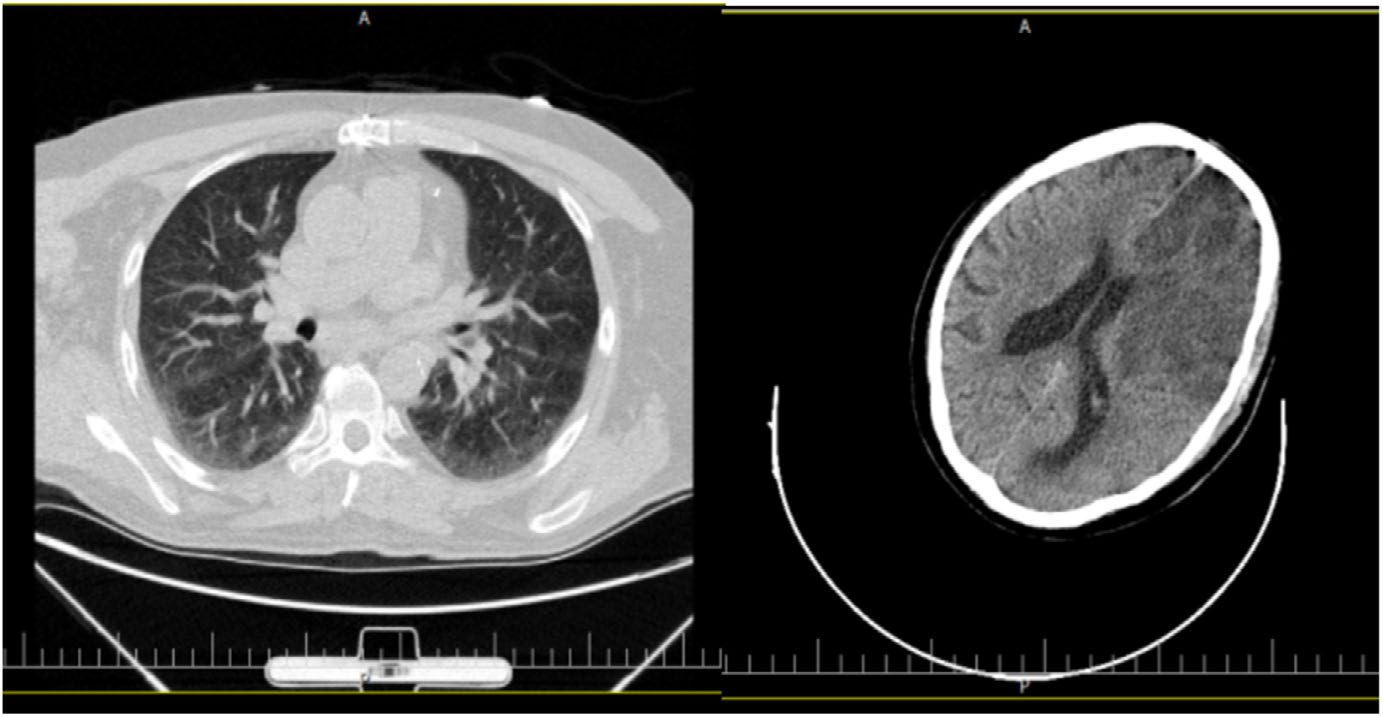
Chest and brain computed tomography.

Blood samples revealed leukocytosis (17,000/μL) with a vital component of granulocytosis but no eosinophilia, platelet counts 266,000 cells/mm3, Hb 11.5 g/dL, s.Cr2.18 mg/dL, and BUN 30 mg/dL. Other significant laboratory results were INR 1.11, aPTT 21 s, ESR 46 m/h, and CRP 29 mg/L. Aside from this, the clinical laboratory data were unremarkable. The chest computed tomography (CT) without contrast agent showed gravitational ground‐glass opacity. A brain CT scan without a contrast agent was performed within 50 min of the patient's arrival and revealed ischemia apparently (Figure 1).

## 3 Method

Upon the patient's arrival, the clinical evaluation of cerebrovascular accident (CVA) and sepsis workup was completed. He was placed on broad‐spectrum intravenous injection antibiotics: 25 mg/kg vancomycin followed by 1000 mg every 12 h and meropenem 1000 mg every 12 h for management of sepsis. The interval between the injection of vancomycin and meropenem was about 30 min.

Due to the absence of any contraindications, rTPA (Actilyse 50 mg, Boehringer Ingelheim, Batch#905254) was commenced (within less than 4.5 h of symptoms onset) for the management of CVA with a standard dosing of 0.1 mg/kg over a minute and 0.8 mg/kg for a 1‐h infusion via the cubital vein of the right arm, without any immediately apparent complications.

Approximately 60 min after the end of the rTPA infusion, a small number of localized vesicles appeared at the injection site. Neither he nor his family had a history of these vesicles.

**FIGURE 2:**
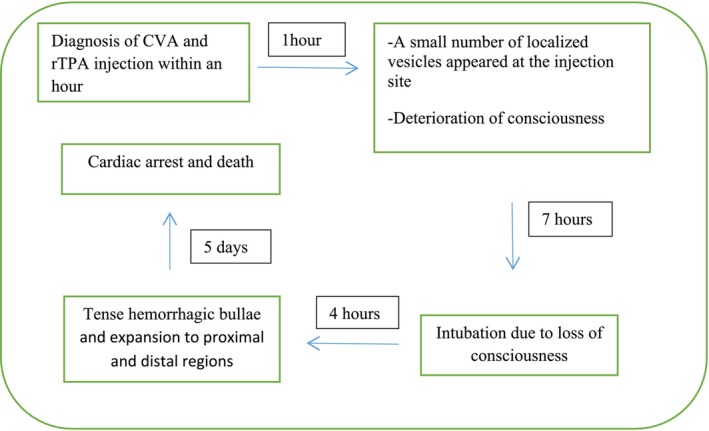
Timeline of clinical events: Initially, after the diagnosis of CVA, vancomycin and rTPA were administered. After 1 h, small vesicles appeared, and the patient's level of consciousness deteriorated. Seven hours later, he was intubated. After 4 h, hemorrhagic bullae expanded. Five days later, he died due to cardiac arrest.

**FIGURE 3:**
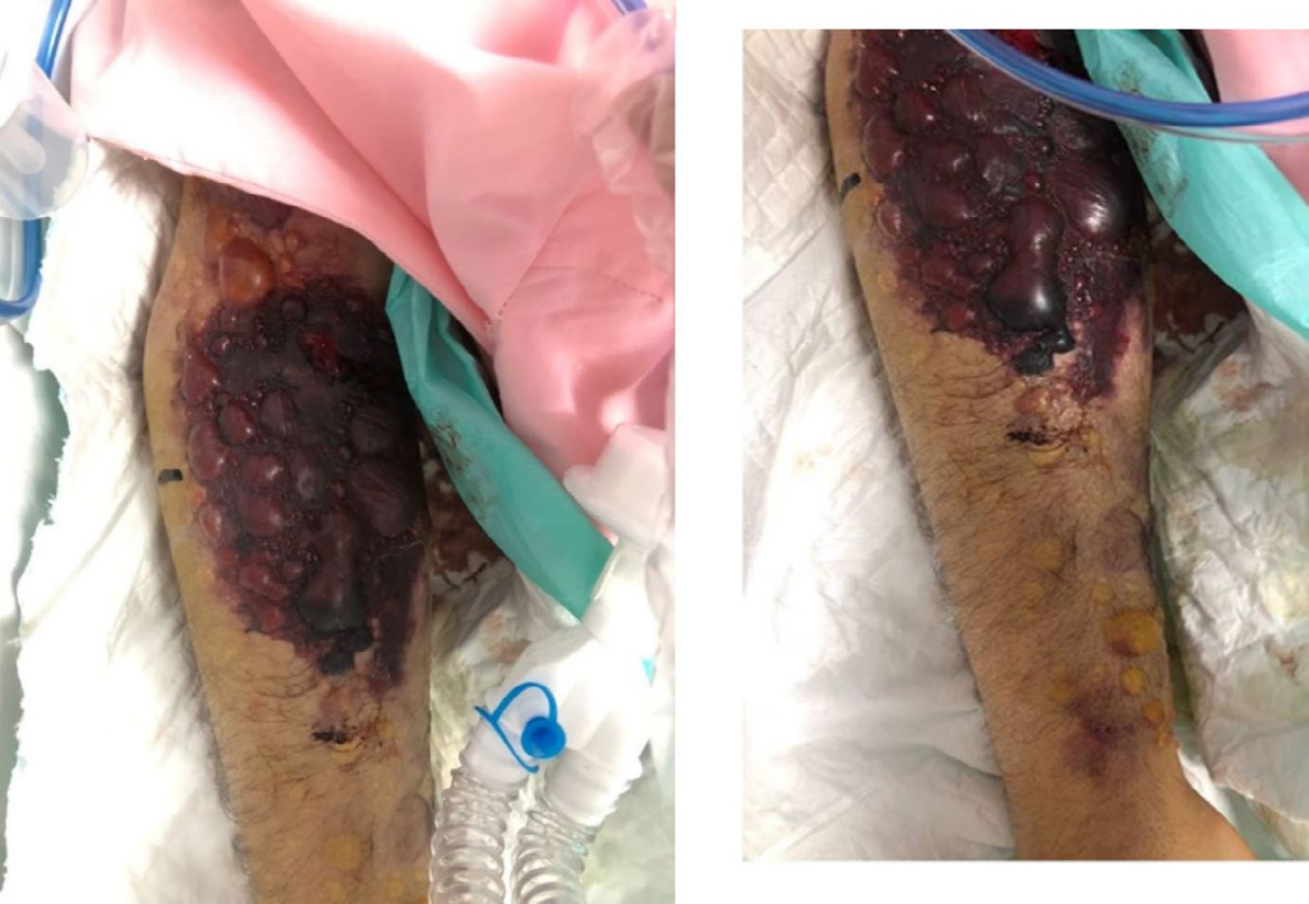
Hemorrhagic bullae associated with ecchymosis on the injection site after 18 h of r‐TPA infusion completion.

The patient's consciousness deteriorated progressively, and he was transferred to the intensive care unit, and intubation was performed within 7 h of symptoms onset. The blister's size increased and gradually became tense hemorrhagic bullae and expanded to proximal and distal regions of the upper limb within 13 h of injection initiation (Figure 2). Furthermore, several significant ecchymoses were observed under the blisters (Figure 3). The diagnosis of hemorrhagic bullae was made by visual inspection within a dermatology consultation. There was no sign of mucosal membrane involvement. Angioedema and bronchospasm were not developed, and fluctuation in blood pressure was not expressed. Nikolsky sign was negative.

**FIGURE 4:**
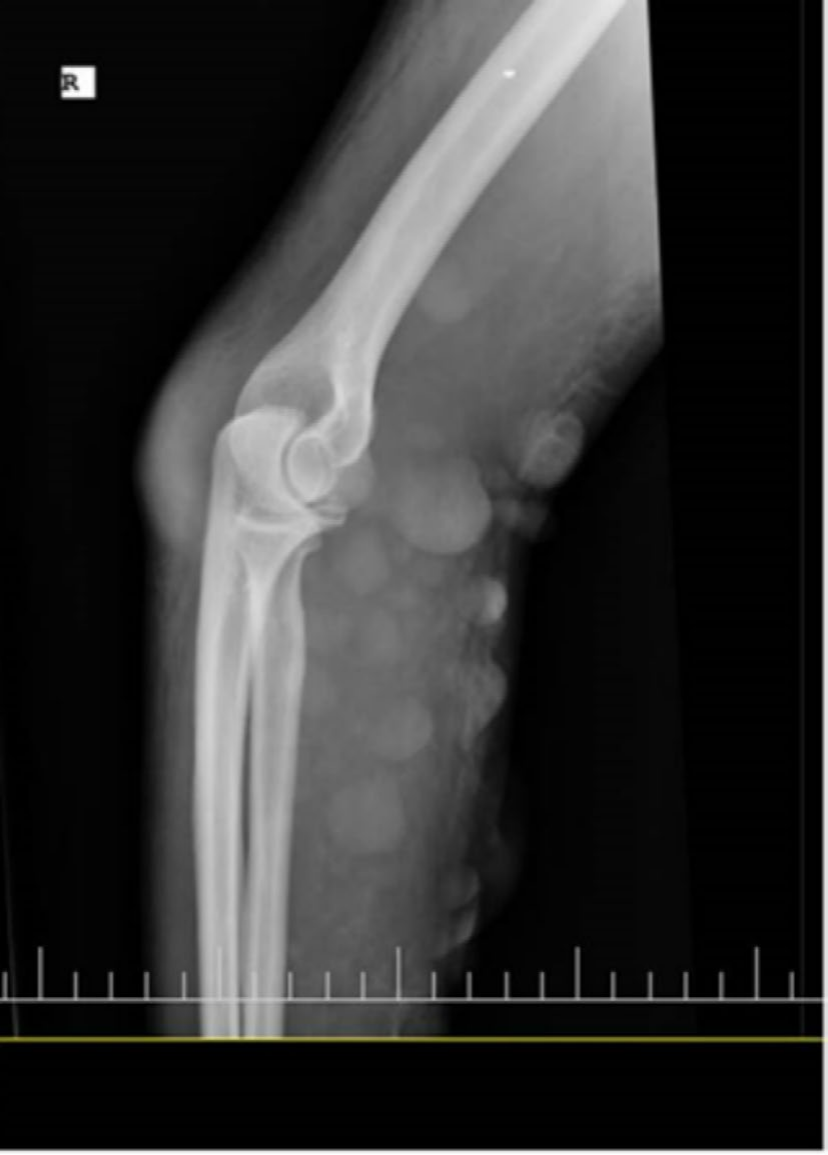
Radiography of the right upper limb demonstrated no evidence of fracture.

The right radial and ulnar pulses were found detectable on examination. The right brachium and antebrachium were swollen, and muscle rigidity was not found. His family did not mention prior exposure to trauma or fracture in the affected site, and the right upper limb radiography was without abnormalities (Figure 4).

The orthopedic surgeon measured the compartment pressure by using a handheld digital manometer. The device displayed a pressure of 15–20 mmHg in line with the blood pressure of 130 over 80 mmHg simultaneously. Eventually, the probability of acute compartment syndrome or necrotizing fasciitis was ruled out. Ultrasound and radiology did not show sub‐tissue involvement.

Blood cultures (aerobic and anaerobic) from two different venipuncture sites and urine cultures were negative. His renal function declined during treatment. On day 3, the level of vancomycin was checked, which was 30mcg/mL.

## 4 Conclusion and Results

Ultimately, after 5 days of admission, he died due to cardiac arrest, while the cutaneous lesions neither improved nor aggravated, and Dermatologists had waited to stabilize the hemodynamic situation to perform a biopsy and manage the lesions.

## 5 Discussion

This case involved an elderly patient with polypharmacy who suffered from sepsis. After the administration of different medications, a hemorrhagic bulla occurred as an adverse drug reaction.

Hemorrhagic bullae have a wide range of differential diagnoses that include immune‐mediated disorders (leukocytoclastic vasculitis, atypical bullous pemphigoid, epidermolysis bullosa acquisita, pemphigus vulgaris, etc.), blood‐based (henoch‐schonleinpurpura, porphyria cutaneatarda), drug or toxin‐mediated (heparin‐induced skin necrosis, warfarin necrosis), infectious (hemorrhagic spider bites, necrotizing fasciitis), and mechanical (bullous hemorrhagic cellulitis, varicella‐zoster virus, friction blood blister) [4].

Bullous hemorrhagic dermatosis is a rare, non‐immune, cutaneous adverse reaction of heparin‐based anticoagulants, characterized by multiple intra‐epidermal hemorrhages distant from the injection site. This complication is most commonly reported with enoxaparin and occurs on average 7 days after drug exposure. This reaction's exact mechanism is unknown [4, 5, 6].

Although the exact risk factors of hemorrhagic bullae are unknown, it was observed that this adverse reaction mostly happens in elderly male patients—also, 42% of patients who have diabetes.

Regardless of whether the suspicious medicine is discontinued, hemorrhagic bullae resolve spontaneously. When the drug was stopped, the shortest duration of lesion resolution was 2 days. When the medication wasn't suspended, the fastest time of lesion resolution was 3–4 days; in most cases, it was resolved after 2 weeks or longer [7]. In our case, any change in the lesions wasn't observed after 5 days. Although the appearance of our case lesion is similar to bullous hemorrhagic dermatosis, there are differences in the onset of the lesion and the site of the lesion.

In this case, antibiotics such as meropenem and vancomycin were administered. There have been no reports of hemorrhagic bullae induced by meropenem, and based on its mechanism of action and the absence of any changes in the lesion despite continued administration, we do not believe this adverse reaction was due to the administration of meropenem.

But vancomycin and rTPA could potentially cause this adverse reaction.

**FIGURE 5:**
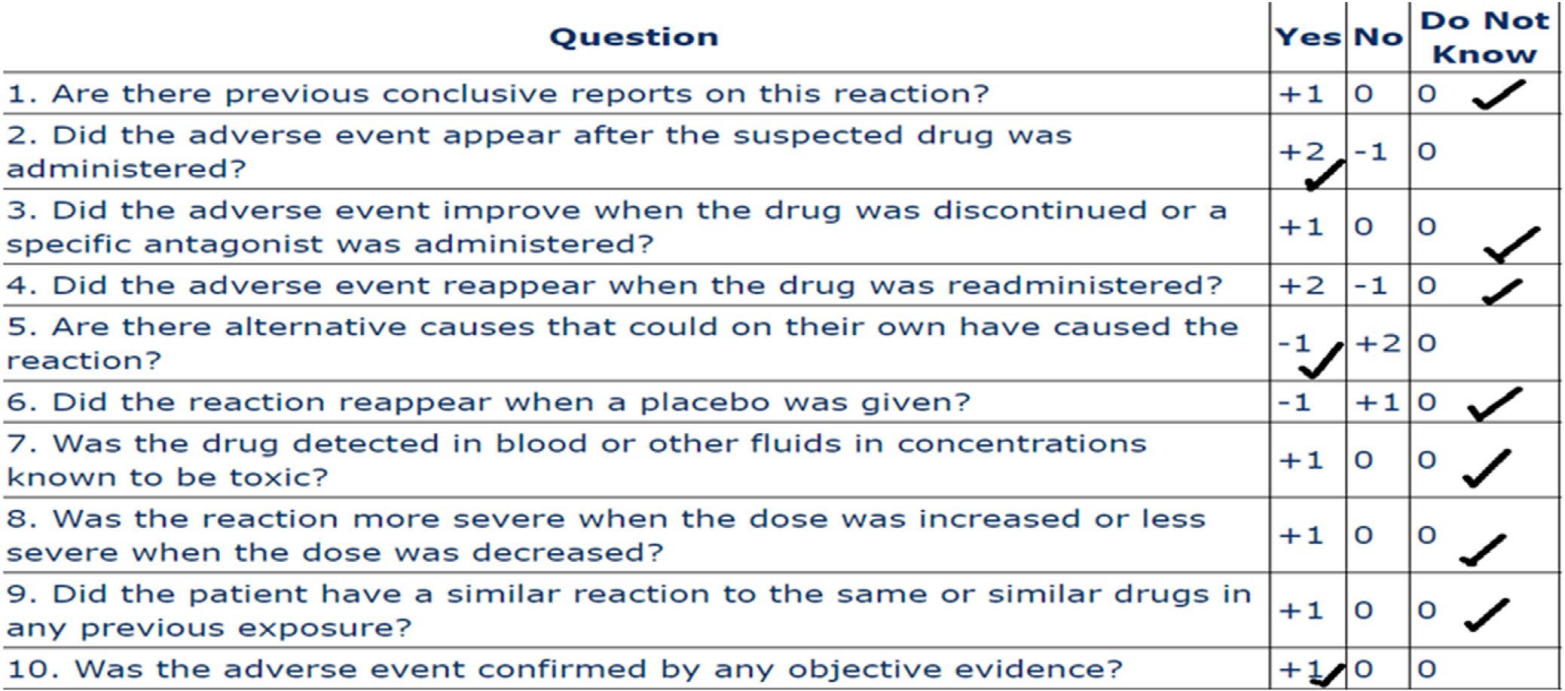
Assessment of ADR by Naranjo scale for rTPA.

The use of fibrinolytic drugs is observed frequently in the emergency department, and hemorrhage is a significant adverse effect of them [8, 9]. To our knowledge, reported cases of fibrinolytic drugs, mainly rTPA‐induced hemorrhagic bullae, have not been described yet, and they would be sporadic. According to the Naranjo adverse drug reaction probability scale, rTPA is a possible reason for hemorrhagic bullae in our case (Naranjo Scale score = 2) (Figure 5).

Another possible reason for this ADR was vancomycin. Vancomycin is a common reason for drug‐induced linear IgA bullous dermatosis. The mean age of patients is 66 [8] years, and men are more likely than women [8]. These lesions have different features, such as hemorrhagic bullae, papules, and vesicles, which are mostly located on extremities, soles, trunk, and appear within 24 h to 15 days after the first dose of vancomycin and resolve after drug discontinuation. In one case, vancomycin didn't stop and continued for 3 weeks, which led to the development of new lesions, and after discontinuation of vancomycin, lesions were dissolved [10]. The diagnosis of linear IgA bullous dermatosis is made based on direct immunofluorescence microscopy testing and observation of linear deposition of IgA along the basement membrane zone [9, 11, 12].

**FIGURE 6:**
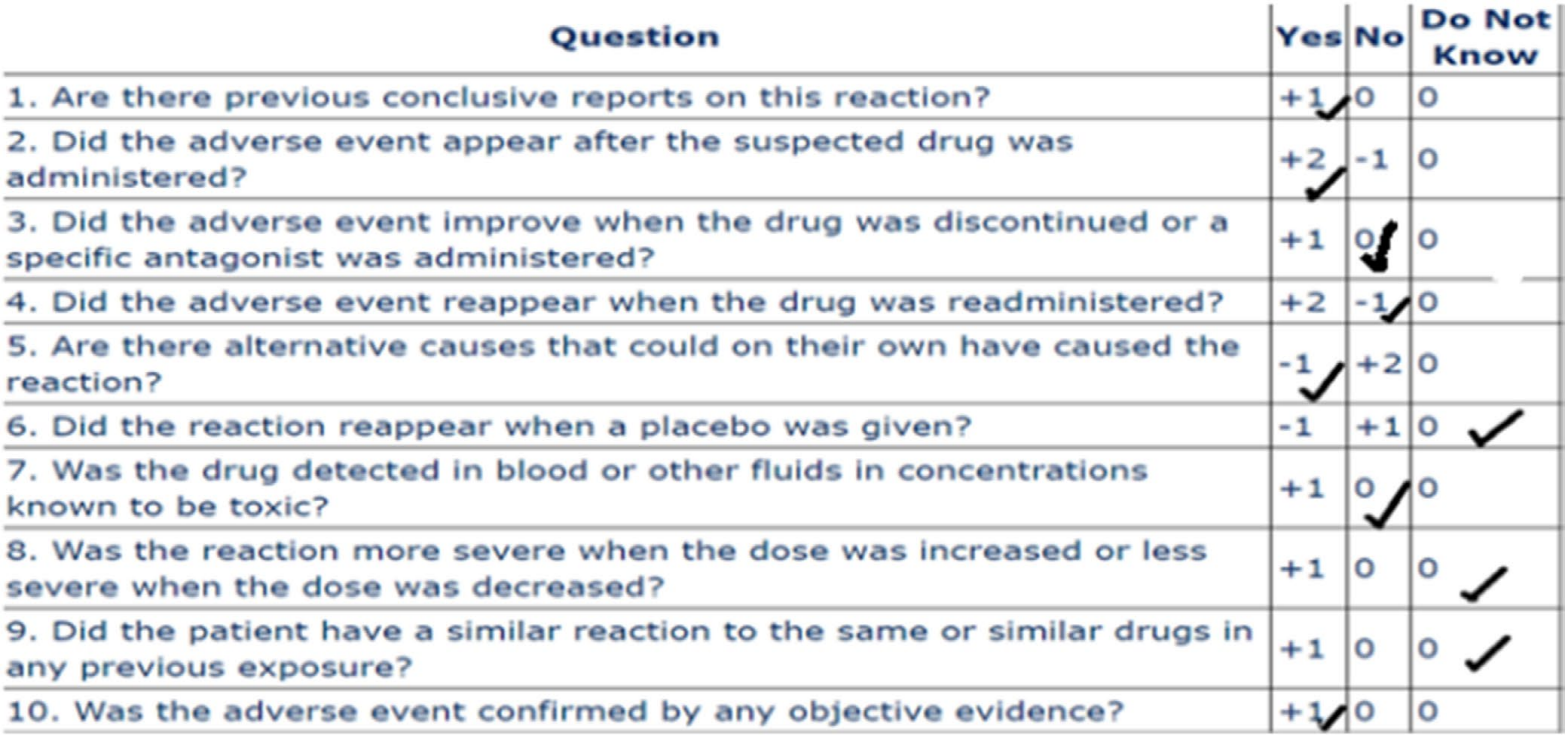
Assessment of ADR by Naranjo scale for vancomycin.

In our case, lesions appeared less than 24 h after the first dose of vancomycin at the injection site. Due to the deterioration of the patient's situation and the clinical requirement to vancomycin, the medicine wasn't discontinued and just the injection sitse was shifted, but no change in the lesion was observed. Also, due to the lack of a biopsy and immunofluorescence test, deposition of IgA was not established. Finally, based on The Naranjo score scale, vancomycin can be the possible reason for this ADR (Score = 2) (Figure 6).

The Naranjo scale is a commonly used system for assessing the relationship between a medication and an ADR. However, it has some limitations. For instance, in cases where there is no prior report of the ADR, the scale may lead to an underestimation of the relationship, so it is not a precise system for evaluation in this situation. Also, rTPA was used as a single dose, and factors like readministration and dose changes were not applied, which could lead to a misleading decrease in the score.

In this case, although the Naranjo score is low and both medications have the same score, based on the mechanism of action and clinical reasoning, the possibility of occurrence of this ADR due to rTPA is more than that of vancomycin. Therefore, we are reporting this case to draw the healthcare team's attention to rTPA. We believe that further studies are necessary to determine whether rTPA can cause hemorrhagic bullae or not. We recommend that the healthcare team stop administering the suspicious medication if it is not crucial and monitor the lesion closely.

Finally, this case reveals a unique instance of drug‐induced hemorrhagic bullae and informs the ED healthcare team to take action properly.

## Author Contributions

**Mehrdad Shavandi:** data curation, investigation, methodology, writing – original draft, writing – review and editing. **Zohre Labbani‐Motlagh:** data curation, formal analysis, writing – original draft, writing – review and editing. **Azin Abdollahi:** writing – original draft, writing – review and editing. **Hadieh Tazerouni:** data curation. **Alireza Naserian:** data curation. **Shahideh Amini:** formal analysis, project administration, supervision, writing – original draft, writing – review and editing.

## Consent

Written informed consent was obtained from the patient to publish this report in accordance with the journal's patient consent policy.

## Conflicts of Interest

The authors declare no conflicts of interest.

## Data Availability

Data sharing not applicable to this article as no datasets were generated or analyzed during the current study.
